# Rapidly progressive papulonodular eruption in a febrile patient

**DOI:** 10.1016/j.jdcr.2026.01.052

**Published:** 2026-02-09

**Authors:** Phuong Daniels, Lauren James, Kaitlyn Stocks, Wanli Cheng, Ashley Rice

**Affiliations:** a Department of Dermatology, Campbell University, Buies Creek, North Carolina; b Novant Health Pathology Department, Wilmington, North Carolina; c Department of Dermatology, Sampson Regional Medical Center, Clinton, North Carolina

**Keywords:** anaplastic large cell lymphoma, cutaneous T-cell lymphoma, histopathology, immunohistochemistry, lymphomatoid papulosis, primary cutaneous anaplastic large cell lymphoma, primary cutaneous CD30+ lymphoproliferative disorders

## Case description

**Fig 1:**
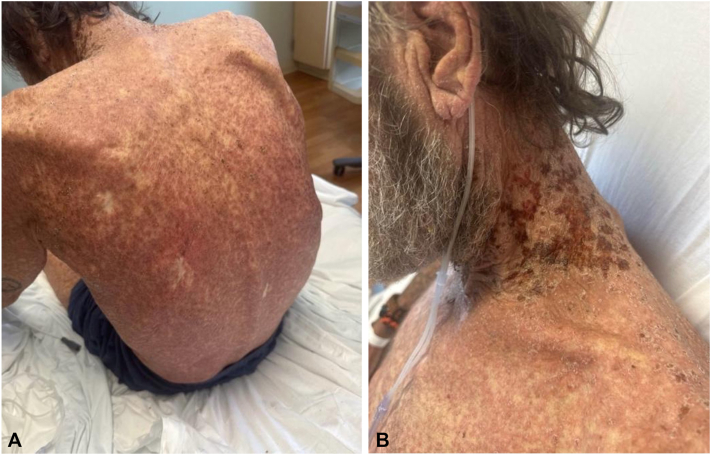
Erythematous papules and nodules diffusely covering the entire body surface **(A)** with honey-crusted lesions involving the left lateral neck **(B)**.

A 63-year-old man with hypertension and type 2 diabetes mellitus presented with a 10-day history of a rapidly progressive, painful, and pruritic papulonodular eruption accompanied by fever, chills, and vomiting. The eruption began on the trunk and quickly generalized to nearly the entire body surface. On examination, he was febrile and appeared ill. Cutaneous examination revealed innumerable erythematous papules and nodules diffusely involving the trunk, extremities, and neck (Fig 1, *A* and *B*). Palpable cervical and axillary lymphadenopathy was present.

**Fig 2:**
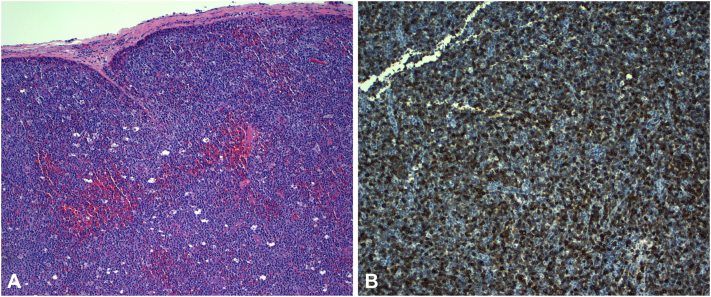
Punch biopsy results showing diffuse atypical lymphocytic proliferation with hematoxylin and eosin (H&E) staining **(A)** and immunohistochemistry positive for CD30 **(B)**.

Punch biopsy demonstrated a dense dermal infiltrate of large atypical lymphoid cells with pleomorphic nuclei (Fig 2, *A*). Immunohistochemistry showed strong, diffuse CD30 positivity and was negative for ALK, with expression of CD3, CD4, CD5, EMA, and a high Ki-67 proliferation index (Fig 2, *B*). Given the fulminant clinical course and systemic symptoms, staging was pursued. Imaging revealed widespread lymphadenopathy and visceral involvement. Excisional lymph node biopsy demonstrated sheets of large atypical CD30^+^ lymphoid cells, confirming systemic disease. The patient rapidly deteriorated and died shortly thereafter.

## Question: What is the most likely diagnosis?

**A.** Lymphomatoid papulosis
**B.** Primary cutaneous anaplastic large cell lymphoma
**C.** Systemic anaplastic large cell lymphoma with secondary cutaneous involvement
**D.** Diffuse large B-cell lymphoma
**E.** Peripheral T-cell lymphoma, not otherwise specified

Correct answer: **C.**

## Discussion

CD30^+^ cutaneous lymphoproliferative disorders range from lymphomatoid papulosis (LyP) to primary cutaneous anaplastic large cell lymphoma (pcALCL) and share substantial histopathologic overlap with systemic anaplastic large cell lymphoma (ALCL) involving the skin.^1^ Accurate classification requires careful clinicopathologic correlation and systemic staging.^2^

Primary cutaneous ALCL typically presents as a solitary or localized tumor or grouped nodules, follows an indolent course, and has a favorable prognosis.^1^^,^^2^ In contrast, systemic ALCL commonly presents with constitutional symptoms, lymphadenopathy, and visceral involvement and may secondarily involve the skin.^3^ Although both entities may demonstrate sheets of large atypical CD30^+^ lymphoid cells and may be ALK-negative, clinical behavior and disease distribution are critical for distinction.

ALK expression further refines classification and prognosis in systemic ALCL. ALK-positive disease usually affects younger patients and is associated with a more favorable outcome, whereas ALK-negative disease typically occurs in older adults and follows a more aggressive clinical course.^3^ Importantly, primary cutaneous ALCL is almost uniformly ALK-negative; therefore, ALK negativity alone does not distinguish between primary cutaneous and systemic disease and must be interpreted in the clinical context.

In this patient, the hyperacute onset, rapid progression over days, fever, diffuse cutaneous involvement, and early nodal and visceral disease are incompatible with pcALCL or LyP and strongly support systemic ALCL with secondary cutaneous involvement.^1^, ^2^, ^3^ LyP is characterized by recurrent, self-healing papules and nodules that spontaneously regress over weeks and follow an indolent course, which is discordant with this presentation.^1^ Diffuse large B-cell lymphoma is excluded by the T-cell immunophenotype, while peripheral T-cell lymphoma, not otherwise specified typically lacks uniform CD30 expression.

Systemic ALCL may rarely present with prominent cutaneous findings, leading to diagnostic uncertainty when skin involvement precedes systemic recognition.^4^ This case reinforces a central teaching point: primary cutaneous CD30^+^ lymphoproliferative disorders are diagnoses of exclusion and require absence of systemic disease.^1^, ^2^, ^3^ The presence of constitutional symptoms, rapidly progressive lesions, lymphadenopathy, or visceral involvement should prompt immediate staging to evaluate for systemic lymphoma.

### Declaration of generative AI and AI-assisted technologies in the writing process

No artificial intelligence tools were used in the writing, analysis, or preparation of this case report.

## Conflicts of interest

None disclosed.
